# Prevalence of Overweight Among Adult Women of A Metropolitan

**DOI:** 10.31729/jnma.5190

**Published:** 2020-07-31

**Authors:** Ashmita Nepal, Urusha Karki, Lisasha Poudel, Bibek Rajbhandari, Shreejana Wagle

**Affiliations:** 1 Manmohan Memorial Institute of Health Science, Kathmandu, Nepal; 2 Central Department Tribhuwan University, Kathmandu, Nepal; 3 Nepal Institute of Development Studies, Kathmandu, Nepal; 4 Nepal Police Hospital, Kathmandu, Nepal; 5 School of Health and Allied Sciences, Pokhara University, Kaski, Nepal

**Keywords:** *adult women*, *Nepal*, *overweight*, *obesity*, *prevalence*

## Abstract

**Introduction:**

Globally, Overweight has reached epidemic proportions. It is an excessive accumulation of fat that may impair health. Overweight and obesity area major contributor to the global burden of non-communicable disease. The prevalence of overweight is commonly assessed by using Body Mass Index (BMI), where overweight is indicated by BMI greater or equal to 25. Overall, about 13% of the world's adult population was obese in 2016, among them women were more affected than men. Overall,female population is higher than male in Nepal and concentrating on Kaski district female population are significantly more than that of male as per population census of 2011. The objective of this study was to find out the prevalence of overweight among adult women.

**Methods:**

This was a cross-sectional descriptive study, conducted among calculated sample size of 185 adult women of Lekhnath Metropolitian of Kaski district, over six months' period. Sampling technique was proportionate random sampling. Ethical approval was taken from Institutional Review Board (Ref no. 40/74/75). Anthropometric measurement was taken to calculate BMI. Collected data was entered in Epi data, which was then exported to SPSS version 20 for further analysis. Descriptive statistics was reported for demographic, socio-economic and various overweight related factors of the respondents as frequencies and percentage.

**Results:**

Out of 185 adult women, 69 (37.3%) of them were overweight, 30 (16.2%) of them were obese, and central obesity was seen among 97 (52.4%) women at 95% C.I.

**Conclusions:**

The finding of this study shows prevalence of overweight and obesity was high. Regular Physical exercise and balanced diet should be followed to prevent overweight and non-communicable diseases.

## INTRODUCTION

Overweight is defined as abnormal or excessive fat accumulation in the body which are major cause of non-communicable diseases.^1^ Prevalence of overweight and obesity is increasing day by day in many low and middle-income countries across the world.^2^ It is one of the most serious public health challenges of the 21st century that contribute to a major risk for serious diet-related chronic diseases (i.e., cardiovascular disease, type 2 diabetes, hypertension and stroke, and certain forms of cancer), physical disabilities, musculoskeletal disorders, asthma, predisposition to some infections, and economic consequences.^3–6^ Overweight and obesity increases health risk, leading to substantial morbidity and disability, impaired quality of life, increased mortality.^7–9^

BMI is a simple index of weight-for-height that is commonly used to classify overweight and obesity in adults.^8^ BMI adds the most practical population-level evaluation of overweight since it is same for both sexes and for all ages of adults. However, it should be advised a rough estimation as BMI may not coincide to the equivalent scale of fat in diverse population.^1^

The objective of this study was to find out the prevalence of overweight among adult women residing in Lekhnath Metropolitian City, Pokhara.

## METHODS

A descriptive cross-sectional study was conducted among adult women of Lekhnath Metropolitan city, Pokhara, Nepal. Data were collected fromJuly to December 2017.

Ethical approval was taken from Institutional Review Board (Ref no. 40/74/75). Permission letter was obtained from the Pokhara Lekhnath Metropolitian office to carry out the study. Informed consent was taken from the Participants and the written consent form was read out to most participants.

The study area was Pokhara Lekhnath Metropolitan,Kaski district, which is located in province no.4 of Nepal. Study population was Adult women aged 18-64 residing in Pokhara Lekhnath Metropolitan. In this area most of the people are following sedentary lifestyle which is risk factor for overweight. Adult women of age group 18-64 years who are permanently residing in Pokhara Lekhnath Metropolitan were included. Participants who were pregnant, who refused to participate and had physical deformities and serious illness were excluded.

Sample Size Calculation

The sample size was determined using the formula, (n) = Z^2^ pq/d^2^

N = z^2^pq/d^2^

where,

z = standard normal variate with value 1.96 at 95% confidence interval
P = prevalence of overweight among adult women in
Nepal(p) = 14% = 0.14^11^
Then, q = 1-p = 1-0.14 = 0.86
d = allowable error, taken as 5%

Here, the total household in Pokhara Lekhnath Metropolitan is 1,05,844 so the population is infinite.

Now for infinite,

$$\begin{array}{ll} \text{The sample size}(\text{n)} & \text{= }{\text{Z}}^{\text{2}}\text{pq}/{\text{d}}^{\text{2}}\\ & \text{= }{(1.96\text{)}}^{\text{2}}*0.14*0.86/\text{0}{\text{.05}}^{\text{2}}\\ & \text{= }185 \end{array}$$

Therefore, sample size was 185.

**Sampling technique:**

Sampling technique used was Proportionate Random sampling that represent the entire population of Pokhara Lekhnath metropolitan city. Following steps were carried out to represent the whole population

**Step 1:** First of all, listing of all thirty-three wards and their respective households of Pokhara Lekhnath Metropolitan city was done. Thirty-three wards of Pokhara Lekhnath Metropolitan city were identified. From each ward the total number of households were listed in sampling frame. That informationwas obtained from the metropolitan office.

**Step 2:** By using simple random sampling technique sixteen wards were excluded and remaining seventeen wards were selected. For the selection of seventeen wards, lottery method was used.

**Step 3:** From each selected ward, required proportion of the sample was chosen for data collection. The following systematic procedure was proposed:

For data collection at first, the center of the sample area was reached and from that center, pencil or bottle was used to spine and the direction shown by the mouth of bottle or tip of pencil was chosen for data collection. Therefore, by using this method required number of samplewas collected.

**Step 4:** The adult woman aged 18-64 years in each selected house were selected for data collection and if there are more than 1 adult women in the selected household then priority was given to adult women of age group 40-59 because greater number of associated factors are present in them. If the selected house does not consist of adult woman aged 18-64 years then other houses were selected using Simple random sampling technique.

**Data collection Technique and Tools**

**Table 1:** Data collection tools and technique

S.N	Tools	Techniques	Purpose
1.	Semistructured questionnaires	Interview schedule	To identify the prevalence of overweight among adult women
2.	Stadiometer	Anthropometric Measurement	To measure the height of the adult women
3.	Adult weighing scale	Anthropometric Measurement	To measure the weight of the adult women
4.	Measuring Tape	Anthropometric Measurement	To measure the waist circumference of adult Women

The translated Nepali version of interview schedule was pretested among 10% of the total sample i.e. 19 adult women aged between 18-64 years of ward no. 10 of Pokhara LekhnathMetropolitan collected data during the pretesting phase was entered in SPSS version20 According to the result of pretesting tool were edited if necessary.

Collected data were entered in EPI-DATA version 3.2. The data from EPI-DATA were exported to SPSS version 22 for further analysis. Categorical variables were described using frequencies and percentages. Descriptive statistics was calculated for most variables using frequencies and summary statistics and percentage to describe the study population. Results were interpreted using tables.

## RESULTS

Out of 185 women, 81 (43.8%) of them had normal BMI, 69 (37.3%) were overweight, 30 (16.2%) obese and 5 (2.7%)underweight. Similarly, 97(52.4%) of women had central obesity (Table 1).

**Table 1:** Distribution of the respondents according to body mass index

Characteristic(n = 185)	n(%)
Body mass index Underweight	5 (2.7)
Normal	81 (43.8)
Overweight	69 (37.3)
Obese	30 (16.2)
Waist circumference	
Normal waist circumference	88 (47.6)
Central obesity	97 (52.4)

Below table revealed the demographic characteristics of the respondents (Table 2).

**Table 2:** Socio-demographic information of respondents

Characteristics	n (%)
**Age(n=185)**	
18-30	81(43.8)
31-39	43 (23.2)
40-49	37 (20)
50-64	24 (13)
Religion(n = 185)	
Hindu	168 (90.8)
Buddhist	5 (2.7)
Christain	12 (6.5)
Ethnicity(n = 185)	
Dalit	25 (13.5)
Disadvantage Janajati	14 (7.6)
Relatively advantage janajati	53 (28.6)
Upper caste group	93 (50.3)
Marital status(n = 185)	
Married	177 (95.7)
Divorced	1 (0.5)
widow	6 (3.2)
Age at marriage (n = 185)	
Early marriage	137 (74.1)
Marriage at proper age	25.9 (48)
No. of children	
Having no children	20 (10.8)
Having less than 2 children	119 (64.3)
Having more than 3 children	46 (24.9)
International wealth index(n = 185)	
Second Quintile	1 (0.5)
Third Quintile	13 (7)
Fourth Quintile	51 (27.6)
Highest Quintile	120 (64.9)
Literacy status(n = 185)	
Illiterate	26 (14.1)
Literate	159 (85.9)
Highest level of education(n = 159)	
Primary	35 (22)
secondary	69 (43.3)
Higher secondary and above	55 (34.5)
Occupation status(n = 185)	
House maker	84 (45.4)
Farming	16 (8.6)
Business	66 (35.7)
Service	13 (7)
Semiprofessional	6 (3.2)

Table 3 shows that out of 185 respondents, the frequency of food intake for most of the respondents 98 (53%), was 3 times a day with 104 respondents preferring carbohydrate and protein (56.2%). Majority of the respondents were non-vegetarian 163 (88.1%) and mostly consuming meat product 1-3 days a week 69 (42.3%). 135 (73%) respondents consumed vegetable on the regular basis. More than half i.e. 101 (54.6%) of the respondent intake fruits more frequently (≥4 times a week). And, 141(76.2%) respondent consumed junk food (Table 3).

**Table 3:** Dietary behavior of the respondents

Characteristic	n (%)
Frequency of food intake in a day(n = 185)	
2times	16 (8.6)
3times	98 (53)
4times	67 (36.2)
5times	4 (2.2)
Food item you prefer most(n = 185)	
Protein	12 (6.5)
Carbohydrate	38 (20.5)
Fats	16 (8.6)
Both carbohydrate and fats	14 (7.6)
Both carbohydrate and protein	104 (56.2)
Fats and protein	1 (0.5)
Vegetarian(n = 185)	
Vegetarian	22 (11.9)
Non-vegetarian	163 (88.1)
Consumption of meat and meat product(n = 163)	
Regularly	37 (22.6)
sometimes(1-3days a week)	69 (42.3)
Once a week	35 (21.4)
Once in 4 days	7 (4.2)
Once a month	15 (9.2)
Consumption of vegetable(n = 185)	
Regularly	135 (73)
sometimes (1-3 days a week)	50 (27)
Consumption of Fruits (n = 185)	
>= 4times a week	101 (54.6)
<4 times a week	84 (45.4)
Consumption of junk food(n = 185)	
Yes	141 (76.2)
No	44 (23.8)
Frequency of intake of junk food (n = 141)	
Daily	31 (21.9)
sometimes	98 (69.5)
often	12 (8.5)

Table 4 shows that out of 185 respondents, only 18 (9.7%) of the respondent performed adequate level of hard work. Majority of the respondent performed adequate level of moderate work 96 (51.9%), adequate level of light work 177 (95%) and passive entertainment activity 132 (71.4%). More than half i.e. 109 (58.9%) of the respondent did nothing regarding fitness. Similarly, 144 (77.8%) of the respondent did not consume alcohol in past six month. Frequency of alcohol consumption for most of the respondent was once a month 15 (36.5%). Majority of the respondent did not smoke cigarette 171 (92.4%). Frequency of smoking cigarette for most of the respondent was on regular basis 12 (85.7%) (Table 5).

**Table 4:** Health risk factors of the respondents.

Characteristic	n(%)
Hard work(n = 185)	
Low hard work	167 (90.3)
Adequate level of hard work	18 (9.7)
Moderate work(n = 185)	
Low moderate work	89 (48.1)
Adequate level of moderate work	96 (51.9)
Light work (n = 185)	
Low light work	8 (4.3)
Adequate level of light work	177 (95.7)
Leisure time utilization(n = 185)	
Moderate work	20 (16.2)
Light work	23 (12.4)
Passive entertainment activity	132 (71.4)
Steps regarding fitness(n = 185)	
Daily exercise	46 (24.9)
Dancing	6 (3.2)
Yoga	24 (13)
Nothing	109 (58.9)
Consumption of alcohol in past six month(n = 185)	
Yes	41 (22.2)
No	144 (77.8)
Frequency of alcohol consumption(n = 41)	
Regularly	6 (14.6)
2-3 days in a week	4 (9.7)
once in a week	8 (19.5)
once in a month	15 (36.5)
Sometimes	8 (19.5)
Smoking cigarette in past six month(n = 185)	
Yes	14 (7.6)
No	171 (92.4)
Frequency of smoking(n = 14)	
Regularly	12 (85.7)
2-3 days in a week	2 (14.2)

## DISCUSSION

The prevalence of obesity is increasing rapidly in both developed and developing countries. It has reached epidemic proportions globally, and evidence suggests that the situation is likely to get worse especially among women.^12^

This study showed the prevalence of overweight and obesity i.e. 37.3% and 16.2% respectively which was more than the study conducted in China among adult women that showed 30.2 % overweight and 12.8% obesity.^13^

Another study conducted in Greece showed that the prevalence of overweight and obesity which was 31% and 15%.^14^

Similarly, a study conducted in Nigerian population showed the prevalence of overweight and obesity which was found to be 29.8% and 36%.^15^

Similarly the study conducted in China, showed the prevalence of central obesity i.e. 37.6% which was less than our study that showed 52.4% of central obesity.^16^ Another study conducted in Greece found that the central obesity prevailed in women was 43%.^14^ Likewise the study conducted in Argentina found the central obesity with 54.8% which was similar to our study.^17^

As for the limitation, our studyexplored prevalence of overweight among adult women of Pokhara Lekhnath Metropolitan of Kaski district, therefore the findings of this study could not be generalized.

## CONCLUSIONS

As per findings of this study, women must be engaged in moderate physical activity, for at least thirty minutes or more on preferably all days of week. Women should reduce the amount of total simple carbohydrate, fat intake to less than one fourth of total calories intakethat helps to prevent unhealthy weight gain in the adult population. Balanceddiet should be followed to protect individual against overweight, as well as non-communicable diseases.

## Conflict of Interest

**None.**
